# Dental Pulp Vascular Response to Early Stages of Caries

**DOI:** 10.1016/j.identj.2024.05.005

**Published:** 2024-06-08

**Authors:** Aljaž Golež, Ksenija Cankar, Aleksandra Milutinović, Lidija Nemeth, Ana Tenyi

**Affiliations:** a University of Ljubljana, Medical Faculty, Institute of Physiology, Ljubljana, Slovenia; b University of Ljubljana, Medical Faculty, Institute of Histology and Embryology, Ljubljana, Slovenia; c University of Ljubljana, Medical Faculty, Department of Dental Diseases and Normal Dental Morphology, Ljubljana, Slovenia

**Keywords:** Dental pulp, Caries, Laser Doppler flow, Vitality test, Volume density of blood vessels, Collagen fibres

## Abstract

**Objectives:**

During caries progression, dental pulp is increasingly pathologically affected. Since the accurate assessment of pulp is of vital importance in clinical decision-making, this study aimed to evaluate pulpal condition in the early stages of caries via laser Doppler (LD) flowmetry and histologic analysis and determine their agreement.

**Methods:**

Fourteen patients with severe dental crowding were included. Prior to extractions and orthodontic treatment, dental pulp condition of 52 premolars was evaluated via LD flowmetry. Teeth were assessed for the presence of caries and lesions were graded according to the International Caries Detection and Assessment System (ICDAS). After extractions, teeth were split and histologically stained for endothelial cells with anti–von Willebrand factor and Movat pentachrome for collagen. Volume densities of vessels (V_vasc_) and collagen were calculated.

**Results:**

There was a significant negative correlation between LD flow and V_vasc_ of the dental pulp with ICDAS grade. Pulpal LD flow and V_vasc_ in teeth with the initial lesion were increased, decreasing with progressing stages of caries. A significant positive correlation between the the pulpal LD flow and V_vasc_, and a negative correlation of LD flow with collagen fibre density were noted.

**Conclusions:**

Caries affects the physiology of the dental pulp, initially with increasing vascularity, and decreasing vascularity at later stges of caries progression. Collagen contents increase with grades of ICDAS. LD flow shows good agreement with the histologic constitution of the dental pulp. Use of clinical measurements of pulpal LD flow could provide a good noninvasive indication of pulpal vascular state and its health.

## Introduction

In the clinical environment of the oral cavity, several detrimental factors affect the integrity and activity of vital pulp tissue; amongst these, those of bacterial origin are the most common. Caries is a multifactorial disease, which results in demineralisation of dental hard tissues and cavity formation, and during this process several mediators activate the response of the underlying pulp tissue.^1^

The accurate assessment of pulp vitality in dentistry is of crucial importance because it determines the dentist's decision for either more conservative dental treatment, which includes preservation of tooth vitality, or more radical treatment, such as endodontic treatment. In clinical practice, standard sensibility tests like thermal (usually cold) or electric stimuli are used for such estimation.^2^ These sensibility tests unfortunately contribute only indirect details that are based first on the individual's perceived reaction to the stimuli and second on the clinician's evaluation of the patient's sensitivity perception. In addition, the consequences of analysing the results of the sensibility tests can often yield questionable or indecisive findings, which may not be in accordance with other clinical findings, making diagnosis even more challenging.^3^

The downside of using cold or electric sensibility testing methods is that the evaluation of the genuine pulp vitality condition cannot be estimated, since these methods do not assess pulpal vascular circulation, only its neural response.^4^ Vascular supply of the pulp, in contrast to its innervation, is a more accurate indicator of pulp vitality.^5^ The most prominent clinical tests for monitoring of pulpal vascular state are laser Doppler (LD) flowmetry and pulse oximetry.

In medicine, histopathological reports are used to confirm the presence and type of certain diseases. In dentistry, however, such a method cannot be used to assess the degree of pulp inflammation or the stage of irreversibility of tissue damage for ethical reasons. As the result of a need to destroy the dental hard tissues of the tooth for the pulp tissue to be obtained, such an approach would cause irreparable damage, as it would require the patient to lose their tooth. Consequently, dentists are left to rely on the results of such diagnostic devices, and though the sensitivity of these tests is high, when false-positive or false-negative results occur, consequences can severely affect the tooth prognosis.^6^ A tooth falsely diagnosed as nonvital with an electric pulp tester may undergo unnecessary root canal treatment, whereas one falsely diagnosed as vital may lead to chronic pulpal infection and its sequalae. Furthermore, due to the latest developments in vital pulp therapy and a desire to preserve pulpal vitality, it is of the utmost significance for clinical diagnosis to correspond to the true pulpal condition.^7^

The dental pulp represents a very vascular soft mesenchyme tissue, occupied by smaller blood and lymphatic vessels, nerves, supporting fibres, ground substance, interstitial fluid, odontoblasts, fibroblasts, undifferentiated mesenchymal cells, stem cells, and defence cells such as histiocytes, mast cells, and plasma cells.^8^ Dentin matrix within the pulp tissue is mostly composed of type I collagen, along with type III and type X collagen fibres.^9^^,^^10^

The aim of our study was to determine the accuracy of LD flowmetry as a method of pulp vitality assessment, testing the correlation between the results of LD flow with the volume density of the blood vessels of the same tooth, in relation to the progression of a carious lesion using International Caries Detection and Assessment System (ICDAS) score assessment.

## Materials and methods

### Participants and sample

Fifty-two upper and lower permanent premolars of 14 patients were included in the study. The Slovenian National Medical Ethics Committee approved the study under protocol numbers 0120-415/2020/6 and 0120-659/2016/6; every participant and their parents or guardians (if they were minors) received prior information and signed the informed consent form. The teeth were scheduled for extraction due to orthodontic indication, mostly due to severe dental crowding. Nine patients were female and aged from 12 to 20 years, and the other 5 were male and aged between 13 and 15 years. The numbers of teeth are listed in Table 1. Before extractions, patients were scheduled for clinical examination and assessment of carious lesions on all teeth surfaces using ICDAS score classification.^8^

**Table 1:** Number of extracted teeth included in the study according to the tooth type and root morphology.

Tooth type	Single-rooted	Double-rooted	Σ
**Upper 1st/2nd premolar (N)**	8	20	28
**Lower 1st/2nd premolar (N)**	24	0	24
**Premolars (N)**	32	20	**52**

### LD flowmetry

LD flowmetry is a simple, noninvasive measurement that allows semiquantitative, continuous monitoring of pulpal blood flow in real time and enables the detection of dynamic changes in the blood flow of the tissue, which includes dental pulp.^11^ The measurements of LD flowmetry (PeriFlux P4001 Master/4002 Satellite, Perimed AB) were performed by a single investigator, as described in previous studies.^12^^,^^13^

For the LD flow measurement, patients were placed in a semirecumbent position. During the measurement, the participants were asked to lay still, breathe normally through the nose, and avoid moving any oral or perioral muscles. An angled probe was used for the measurement. It was stabilised on the dental arch and placed on the buccal tooth surface in the gingival third of the tooth crown, where the dental pulp tissue of the tooth is projected.

The value of the pulpal blood LD flow in perfusion units (PU) was registered. LD flow in each participant's tooth was measured for at least 1 minute. An average value and standard deviation of the measurements within the measuring period were subsequently calculated.

### Tissue samples and staining

The preparation process of the extracted teeth was performed as described previously.^14^ Immediately after extraction, one-third of the apical part of the root was separated for better penetration of the fixating suspension into the pulp tissue and fixed in 10% buffered formalin for 24 hours, as it was reported to be a method for achieving the best penetration rate of the fixative.^14^, ^15^, ^16^

After 24 hours, the entire tooth's vertical (longitudinal) split was performed. The halves of the tooth with pulp were reimmersed in 10% buffered formalin for another 48 hours. Then, the pulps were cautiously removed from the dental half, dehydrated in alcohol, immersed in xylene, embedded in paraffin, and cut into 4.5-µm thick longitudinal step serial sections. The step between the 2 sections was 20 μm thick. Sections were stored at room temperature and stained with haematoxylin and eosin. Blood vessels were shown by immunohistochemical labelling of endothelial cells with anti–von Willebrand factor (vWf; Dako Denmark; 1:800).^17^ Collagen fibres were displayed using Movat pentachrome stain, which presented collagen fibres in yellow and ground substance in blue.^18^

### Image analysis and evaluation of the volume density of blood vessels, collagen fibres, and ground substance

Image analysis was performed under a light microscope (Nikon Eclipse E400), using a camera (Nikon Digital Sight DS-M5) and NIS-Elements (version 3, Nikon Instruments, Inc.) documentation computer programme. The measurements were performed on 3 sagittal slices of the central part of the dental pulp at the objective magnification of 40× for blood vessels as well at the objective magnification of 40× for collagen fibres and ground substance in the whole section (crown and root part) of the pulp tissue.

The volume density of the blood vessel lumen, collagen fibres, and ground substance was stereologically analysed, respectively, using Weibel's test system as described previously.^19^^,^^20^

### Statistics

Statistical analysis was performed using SigmaPlot 14.0 software (Systat). The sample size was determined, using the power of the study at 0.8 and *P* value significance at <.01. The result for the appropriate sample size was N = 19. Our study involved 52 specimens.

Shapiro–Wilk and Brown–Forsythe tests were used to check for normality and equal variance. One-way analysis of variance (ANOVA) was used to test for differences between LD flow values of the groups of teeth with different ICDAS scores. To differentiate groups amongst themselves, the Bonferroni post hoc test was used.

The correlation between the volume density of blood vessels and collagen fibres, and LD blood flow were tested by Pearson coefficient of correlation. *P* values <.05 were considered statistically significant.

## Results

In total, 37 of 52 extracted premolars included in the present study had at least 1 initial carious lesion visible on its surface. When several carious lesions were visible on the same tooth, a code with a higher ICDAS score was noted and included in the statistics. No carious decay greater than ICDAS 3 was observed on any of the extracted teeth. All carious lesions were assessed as an inactive type of ceased progression. The activity of the lesions was assessed according to the visual appearance of the lesion (ICDAS scores), the location of the lesion (in plaque stagnation areas or not in plaque stagnation areas), and the tactile feeling of the lesions.^21^ All teeth irrespective of caries grade were asymptomatic. The distribution of ICDAS findings is presented in Table 2.

**Table 2:** Number of teeth according to the International Caries Detection and Assessment System (ICDAS) score.

ICDAS	Upper 1st/2nd premolar (N)	Lower 1st/2nd premolar (N)	Σ (N)
**0**	8	7	15
**1**	2	5	7
**2**	8	5	13
**3**	10	7	17

### Dental pulp LD flow measurement

ANOVA showed a significant difference in pulpal LD flow amongst the groups of teeth with different ICDAS scores (*P* = .01). Teeth with an ICDAS score of 3 had significantly lower pulpal LD flow values when compared to the teeth with an ICDAS score of 1 (Bonferroni test, *P* = .008). Findings are presented in Table 3.

**Table 3:** Mean laser Doppler (LD) flow values in perfusion units (PU) according to the International Caries Detection and Assessment System (ICDAS) score.

ICDAS	No.	LD flow (PU)
**0**	15	11.034 ± 3.16
**1**	7	13.362 ± 3.21
**2**	13	10.252 ± 3.85
**3**	17	8.689 ± 2.08

Values are mean ± standard deviation.

### Correlation between ICDAS score and pulpal LD flow

A statistically significant negative correlation between ICDAS score and LD flow was observed (*R* = −0.333, *P* = .016; Figure 2).

### Tissue samples

**Fig. 1:**
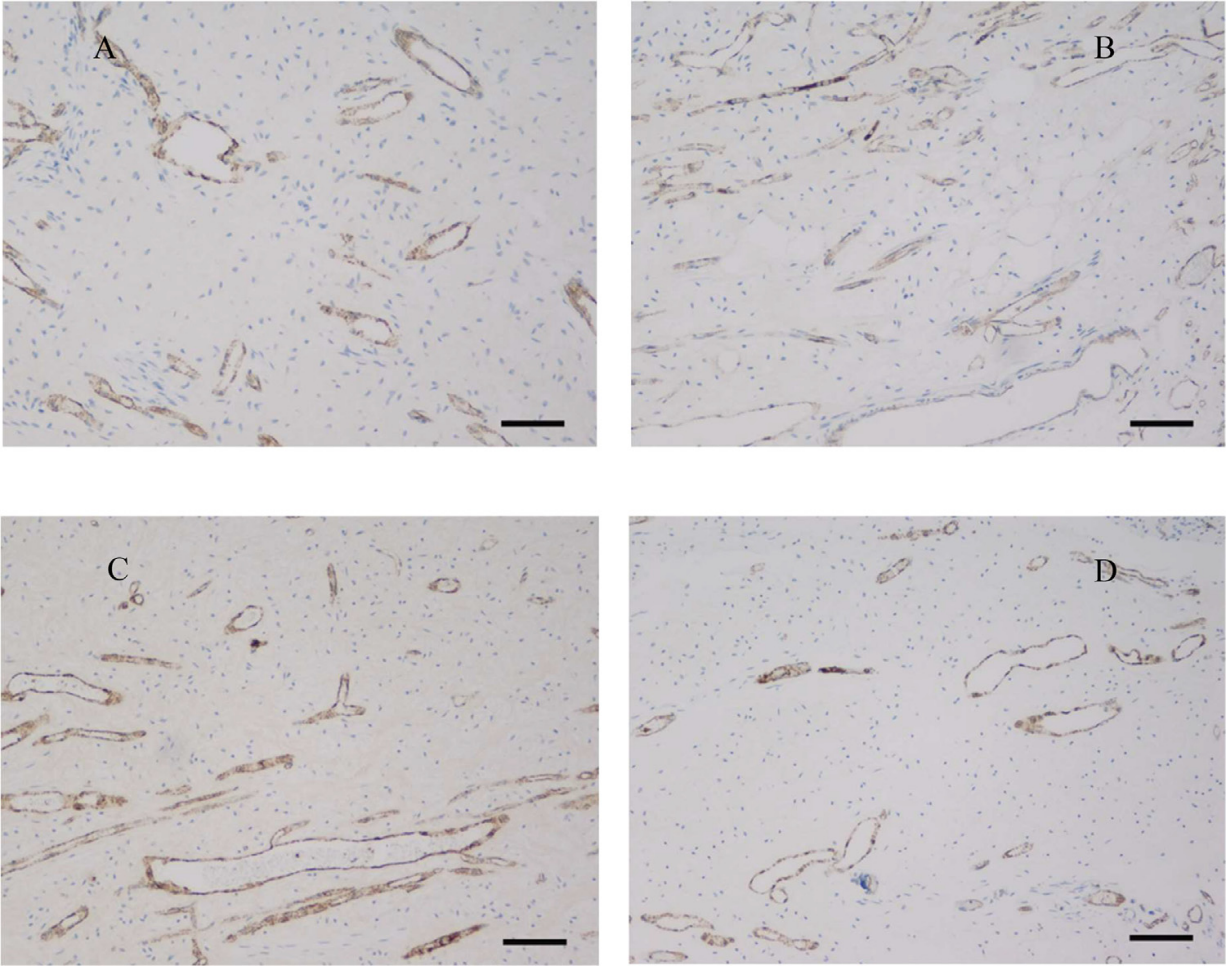
Dental pulp stained with anti–von Willebrand factor (objective magnification 10×, bar = 100 μm). Note that the pulp tissue is significantly more vascularised in the International Caries Detection and Assessment System (ICDAS) 1 group (**B**) in comparison to groups ICDAS 0 (**A**), ICDAS 2 (**C**), and ICDAS 3 (**D**).

Histologic examination of the pulps stained with Movat pentachrome and immunohistochemically for vWf (Figure 1) showed the loose connective tissue in the central part of the pulp. The peripheral part consists of the odontoblastic and subodontoblastic zones, composed of a cell-free (Weil zone) and a cell-rich (Höhl zone) layer. The odontoblastic layer was frequently pulled apart from the dentin during pulp isolation (Figure 3). The pulp tissue had many blood vessels with a thin wall. The main arterioles, venules, and nerves ran aligned with the long axis of the tooth. They were found in the central part of the root canal. Blood vessels branched many times at right angles. Arteries that run from these branches often had a diameter greater than the vessel from which they originated. Transversal branches between vessels were also prominent. In the coronary part of the pulp, the axons and blood vessels extensively branched. The density of the capillary bed and nerve plexus increased towards the upper part of the dental pulp in the crown.

Histologic analysis of vascular volume density values of teeth was grouped according to the ICDAS scores of caries lesions. Vascular volume densities differed significantly amongst the ICDAS scores (ANOVA on ranks, *P* = .023). Vascular volume density was significantly greater in teeth with an ICDAS score of 1 compared to noncarious teeth (ICDAS 0). In addition, teeth with an ICDAS score of 3 had significantly lower vascular volume density compared to teeth with an ICDAS score of 1 (Dunn post hoc test, *P* < .05; Table 4, Figure 1)

**Table 4:** Vascular volume density values according to the International Caries Detection and Assessment System (ICDAS) score.

ICDAS	N	Vascular volume density (mm^3^/mm^3^)
**0**	15	0.219 (25**%** 0.0877, 75% 0.291)
**1**	10	0.301 (25**%** 0.287, 75% 0.323)
**2**	16	0.279 (25**%** 0.192, 75% 0.310)
**3**	17	0.221 (25**%** 0.158, 75% 0.281)

Values are median (25th percentile, 75th percentile).

### Correlation between vascular volume density and pulpal LD flow

**Fig. 2:**
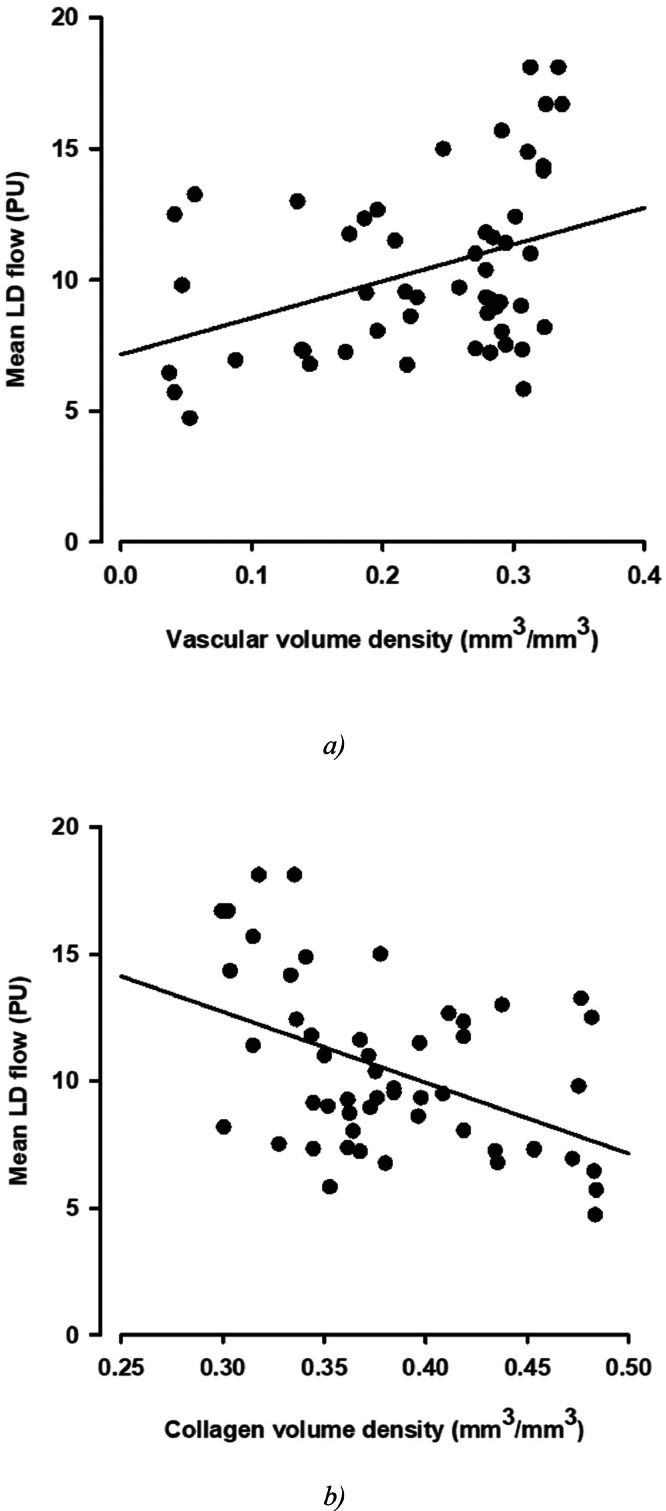
Correlation between pulpal (**A**) vascular volume density and laser Doppler (LD) flow and (**B**) collagen volume density and LD flow.

A significant positive correlation between pulpal vascular volume density and pulpal LD flow was observed (*R* = 0.379, *P* = .000559; Figure 2A). In addition, a significant positive correlation between the vascular volume density of the pulp tissue, including the volume density of ground substance, which represents the pulp tissue containing great amounts of water substance, and LD flow was found (*R* = 0.436, *P* = .001124).

**Fig. 3:**
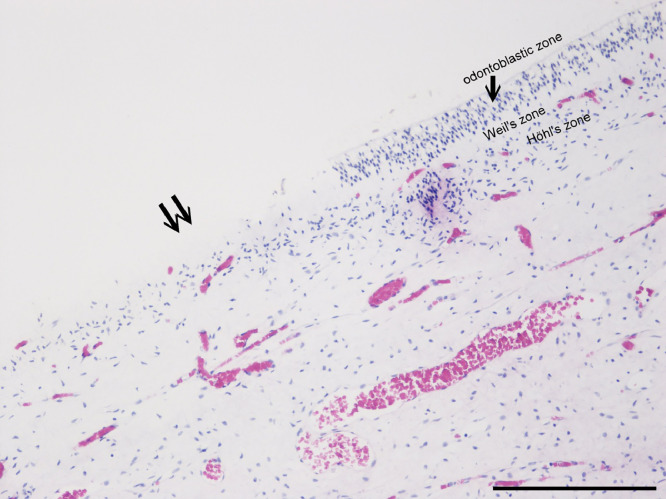
The odontoblast layer and Weil and Höhl zone; note the area where the odontoblast layer was torn off when the pulp was isolated from dentine (↑↑).

A significant negative correlation between the collagen volume density of the pulp tissue, which represents the connective tissue of the pulp, containing less water, and LD flow measured was noted (*R* = −0.456, *P* = .000674; Figure 2B).

## Discussion

In the present study, we found a positive correlation between the volume density of blood vessels and the pulp tissue's LD flow levels, as well as a positive correlation between the volume density of blood vessels and density of ground substance and the pulp tissue's LD flow level independently. Results also showed a negative correlation between collagen tissue density and pulp tissue's LD flow.

In addition, a negative correlation between carious progression assessed by ICDAS classification and carious progression assessed by measurements of blood flow of the pulp using the LD method was observed.^12^ Mean values of LD flow in our study (8–13 PU) were comparable to the values from other studies obtained in children of similar age (7–14 PU).^22^ Because pulpal blood flow is mostly locally controlled, the patient's usual chores, such as exercise, do not majorly affect its values.^23^ Most pulpal LD flow measurement errors can be minimised by restricting movement of the probe, LD flowmetry device, and subject as well as positioning the LD probe to limit signals from other adjacent perfused oral tissues (eg, periodontium, gingiva, oral mucosa). One of the major disadvantages of LD flowmetry is that it is a relatively complex and expensive system available only to larger clinical and research facilities.^24^ It is the most time-consuming amongst the pulpal tests as well.^25^

The dental pulp is well recognised as a very vascular tissue.^26^ It was reported that in young cats, 14% of dental pulp volume is composed of vessels and the average density of the capillary network was found to be 1402 mm^2^.^20^^,^^27^^,^^28^ The characteristics of pulpal vascular bed in our study were: high density, thin walled and narrow diameter vessels. Similar properties have been observed in previous studies.^29^^,^^30^ Significant differences in pulpal blood flow measured with LD flowmetry were noted amongst teeth with different stages of carious lesions according to ICDAS score criteria. Pulpal LD flow in teeth categorised as ICDAS 1 was higher compared to teeth without caries (ICDAS 0). These findings suggest that pulpal response to the infection starts at an early stage, even though the caries lesion is superficial (ICDAS grade 1–3). This result is also in agreement with other studies, which suggested that pulp tissue response starts even at initial carious lesions.^31^^,^^32^ The pulpal reaction to initial caries lesions is likely a nonspecific inflammatory response to localise and limit the noxious agent in the tissue.^29^ Inflammatory response through the release of vasodilation agents (nitric oxide, calcitonin gene-related peptide, platelet-derived growth factor, vascular endothelial growth factor, adenosine, histamine, bradykinin, prostaglandin PGE2) and cytokines, which act on vascular smooth muscle and endothelial cells, activates leukocytes and consequently causes increased blood flow, plasma extravasation, and escape of white blood cells into the pulpal tissue. Release of these factors and vasodilation of pulpal blood vessels were likely associated with an increased pulpal LD flow in very early carious lesions (ICDAS 1).

In contrast, teeth categorised as ICDAS 3 had significantly lower LD flow compared to those categorised as ICDAS 1. With the progression of caries, the inflammatory response is enhanced, which could in turn lead to increased filtration according to the Starling principle.^33^ Since pulpal walls are noncompliant, increased filtration and fluid volume inside dental pulp will result in higher interstitial pressure, which tends to compress pulpal arterioles, decreasing blood flow.^34^^,^^35^

Additionally, bacterial by-products further increase vascular permeability, upregulate pro-inflammatory cytokines (interleukin-1, tumour necrosis factor–alpha, interleukin-6) and affect normal endothelial function (“perturbation”), all of which reduce the pulpal vascular supply further, thus reducing its defensive function.^36^, ^37^, ^38^ To the best of our knowledge, no clinical study has evaluated changes in pulpal blood flow in the early carious lesion. Nevertheless, Nemeth et al^13^ previously found that teeth with advanced caries (ICDAS 6) and teeth with active caries had lower pulpal LD flow, which is in agreement with our findings.

The histologic preparation of tooth samples is often very challenging due to the presence of both mineralised hard tissues and soft tissues. To assess the density of histologic structures in tissue slices, we chose a stereologic method for volume density calculation. Using the same method, Santamaria et al^39^ measured blood vessel volume density in the pulp tissue of rat teeth after orthodontic movement. Their results ranged between 0.07 and 0.1 cubic mm, whereas the average volume density of blood vessels in our study was 0.28 cubic mm. Values between studies may cary due to the difference between animal and human pulp tissue morphology, and hence further research is warranted in this cintext.

A similar relationship to pulpal LD flow was observed between stages of ICDAS and histologically measured vascular volume densities. Noncarious teeth and teeth classified as ICDAS 1 were more densely vascularised compared with later stages of caries progression (ICDAS 3). Histologically observed pulpal vascular volume densities correlated well with clinically measured pulpal blood flow via LD flowmetry, indicating that LD flow does reflect the true condition of the pulpal vasculature. Even though we are the first to describe such a relationship, the results of previous laboratory models corroborate these empirical findings.^40^

Results showed that teeth with carious lesions at higher ICDAS stages had higher collagen volume densities. Because higher-grade caries lesions are chronic conditions that take longer to develop, a greater degree of collagen fibrosis could be expected. As part of inflammatory reaction, enzymes such as matrix metalloproteinase (MMP)–2 and MMP-9; growth factors such as fibroblast growth factor (FGF) and transforming growth factor β (TGF-β); and the transcription factor C-Fos affect migration and proliferation of fibroblasts, which play a role in pulpal extracellular matrix formation and remodelling of collagen fibres in the dental pulp.^41^ If these effects persisted, accelerated fibrotic transformation and tertiary dentine formation would be expected. All these histologic changes in microcirculation, inflammatory infiltration, and collagen deposition the dental pulp that are caused by initial caries did affect the pulpal vitality test using LD flowmetry.

One of the limitations of the present study is that it lacked teeth with more extensive carious lesions (ICDAS 4 and above). Every lesion was inactive, and all participants reported no symptoms, neither pain nor discomfort regarding having a carious lesion; therefore, their pulps could still be considered within the healthy-to-inflamed spectrum. In daily clinical practice, dentists are confronted with much more challenging situations, where pulp might be mild, moderately, or severely inflamed or even partially necrotic.^42^ So far, there are very limited data on pulpal blood flow in various stages of pulpitis, and research in dental pulp during pathologic events could meaningfully expand current understanding.^43^ On the other hand, a study by Nemeth et al^13^ that clinically measured pulpal blood flow via LD flowmetry did include teeth with higher grades of ICDAS carious lesions, whilst Ricucci et al^44^ analysed pulpal vessels with irreversible pulpitis histologically. Their results give insight into the reaction of dental pulp with more advanced caries and irreversible pulpitis, and their findings follow similar patterns observed in our study. The relationship between the clinical status of the pulp and its histologic state was assessed in many studies, and an inconsistent association between histologic pictures and clinical signs was often found.^42^^,^^45^^,^^46^

Nevertheless, the gold standard for assessment of the actual state of pulpal tissue remains the histologic examination of the dental pulp.^4^ Unfortunately, to apply the gold standard, the extraction of the tooth shortly after the use of the diagnostic tests would be necessary, which makes this method inappropriate and unethical, especially when clinical indication suggests that the tooth can be saved. All of these factors limit the sample of teeth available for research purposes.

To clinically determine the condition of pulpal tissue, direct inspection cannot be performed since the pulp tissue is enclosed within the calcified barrier of dentin and enamel. Therefore, dentists must use indirect methods to anticipate pulpal vitality. Our study showed that LD flowmetry correlated well with the gold standard of histologic evaluation of dental pulp vasculature; therefore, in clinical settings, LD flowmetry could provide a reasonably good indication of pulpal vascular state.

## Conclusions

The results of the study confirm that pulpal blood flow using LD flowmetry has a high correlation with the true histological appearance of the tissue Progressive stages of caries, from the early to late stages affect the vascularisation and the collagen content of the pulp.

## Funding

This work was funded by the programme grants of the Slovenian Research Agency, ARRS P3-0019, and the project J3 - 50103. The funder had no role in the design, data collection, data analysis, or reporting of this study.

## CRediT authorship contribution statement

**Aljaž Golež:** Conceptualization, Methodology, Investigation, Formal analysis, Investigation, Resources, Data curation, Writing – original draft, Writing – review & editing, Visualization, Project administration. **Ksenija Cankar:** Conceptualization, Methodology, Software, Investigation, Resources, Writing – review & editing, Visualization, Supervision, Project administration, Funding acquisition. **Aleksandra Milutinović:** Conceptualization, Methodology, Investigation, Resources, Writing – review & editing, Supervision, Project administration, Funding acquisition. **Lidija Nemeth:** Conceptualization, Methodology, Investigation, Resources, Writing – review & editing, Supervision, Project administration, Funding acquisition. **Ana Tenyi:** Conceptualization, Methodology, Investigation, Formal analysis, Investigation, Resources, Data curation, Writing – original draft, Writing – review & editing, Visualization, Project administration.

## Conflict of interest

The authors declare that they have no known competing financial interests or personal relationships that could have appeared to influence the work reported in this paper.
